# Chloroform Hallucination

**Published:** 1869-11

**Authors:** 


### CHLOROFORM HALLUCINATION.

Our readers may remember the shameful sacrifice of a Philadelphia dentist, a few years ago, to the demands of an ignorant rabble. He was sent to the Penitentiary on the unconfirmed testimony of a single individual, against all law and precedent. We never believed him guilty.

*The Eclectic Medical Journal*, of Cincinnati, in some pertinent comments on the subject, reports a case from *The Louisville Journal*, where, in the presence of eight of the most eminent physicians of Louisville—some of whom were well known to the Editor for more than a quarter of a century—a lady, under the influence of chloroform for the removal of a tumor, charged the operator, the moment she recovered, with taking the most unwarranted liberties, and left the room in the utmost indignation. No man of any prudence or common sense ought to administer chloroform, except in the presence of a third person of intelligence and maturity. For ourselves, we prefer to encounter danger with our eyes open.

